# Emerging roles of pterins as signaling molecules in bacteria

**DOI:** 10.1042/BST20250041

**Published:** 2026-06-08

**Authors:** Pierson Rucker, Lauren Augusta, Clay Fuqua, Kylie D. Allen

**Affiliations:** 1 Department of Biochemistry, Virginia Tech, Blacksburg, VA 24061, U.S.A.; 2 Department of Biology, Indiana University, Bloomington, IN 47405, U.S.A.

**Keywords:** Biofilm, c-di-GMP, Proteobacteria, pteridine reductase, Pterins, Signal transduction

## Abstract

Pterins are redox-active biomolecules with well-established functions as enzymatic cofactors in all domains of life. Examples of pterin cofactors include tetrahydrofolate, an essential carrier of one-carbon units, and the molybdenum cofactor (Moco)—composed of molybdopterin bound to molybdenum or tungsten—which supports a wide range of redox reactions for diverse enzymes. Pterins also play important roles outside of enzyme active sites where they serve as antioxidants and immune system modulators. Recently, a new role for pterins in bacteria has emerged through the discovery of a pterin-dependent regulator called DcpA that modulates the cytoplasmic second messenger cyclic-di-guanylate monophosphate (c-di-GMP) levels and biofilm formation in *Agrobacterium tumefaciens*, a model bacterial plant pathogen. The regulatory pathway involves the PruA pteridine reductase and a periplasmic pterin-binding protein termed PruR. This review provides an overview of each of the key players in this pathway and highlights the broad distribution of the respective genes that supports the presence of similar pterin-mediated regulatory pathways in diverse bacteria. In our current model for the *A. tumefaciens* pathway, PruA generates a tetrahydropterin species that is released to the periplasm where it binds to PruR. The pterin-PruR complex then interacts with the periplasmic domain of DcpA to promote c-di-GMP degradation. The decreased levels of cytoplasmic c-di-GMP disfavor attachment and limit biofilm formation. The redox active nature of pterins is likely a central facet of their signaling functions, where the different redox states of a given pterin species may reflect changing environmental conditions.

## Introduction

**Figure 1:**
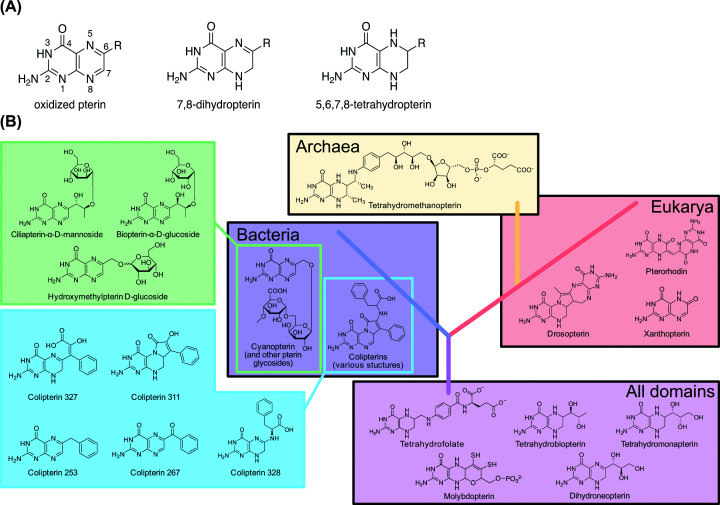
Pterin structures (**A**) Different oxidation states of the pteridine ring system. (**B**) Diversity of pterins across the tree of life. Pterins are shown in the most biologically relevant oxidation state when known. Representative cyanobacterial pterins (Hanaya et al. 2025 [14]) and colipterins (Park et al. 2020 [53]) are highlighted. The colipterins are lablled according to their original identified masses. Colipterin 458 is shown as a representative structure in the core diagram. It is important to note that biopterin is rare in bacteria/archaea and more commonly is synthesized and utilized in eukarya. Tetrahydromethanopterin is present in methanogenic archaea, but not all archaea.

Pterins are ubiquitous biomolecules composed of fused pyrimidine and pyrazine rings (“pteridine”) with amino and keto groups at positions 2 and 4, respectively [1] (Figure 1A). They are defined by the identity of their sidechain at the C-6 position, and some are further modified at the C-7 position. Pterins exist in three major oxidation states: fully oxidized, the partially reduced 7,8-dihydro (H_2_) form, and the fully reduced 5,6,7,8-tetrahydro (H_4_) form [1,2] (Figure 1A). These molecules are found in all domains of life and play diverse roles, ranging from pigments to enzymatic cofactors, with their activities often modulated by their redox state [2,3]. While the specific functions of several biological pterins have been well characterized, the roles of many remain cryptic, partly owing to their broad chemical capabilities.

Pterins were first discovered in butterfly wing extracts [4], later owing their name to the Greek “pteron,” meaning wing [5]. Examination of pigmental pterins in animals revealed many unique structures, which range from single group additions on the pteridine core to additional fused rings, resulting in diverse optical properties [6]. Beyond their initially studied functions as pigments, eukaryotic pterins serve key roles as antioxidants, immune regulators, and enzyme cofactors [7–10]. For example, tetrahydrobiopterin (H_4_BPt) functions with hydroxylase enzymes that catalyze the production of neurotransmitters, as well as nitric oxide synthase, which regulates vascular function through nitric oxide production [10,11]. Bacterial pterins were first described in cyanobacteria [12,13], which produce several pterin glycosides at high levels [14] (Figure 1B). Although the roles of these cyanobacterial pterins are still not completely understood, they appear to play roles in light protection [15,16] and mediating phototaxis [17], and have received interest as bioindicators in environments prone to algal blooms [18,19].

Molybdopterin (MoPt) and tetrahydrofolate (H_4_folate) are pterin cofactors with widely conserved structures and functions across all domains of life (Figure 1B). MoPt is a pyranopterin-dithiolate that coordinates molybdenum (or, less frequently, tungsten), along with cysteine, serine, or aspartate residues in the active site, together comprising the molybdenum cofactor (Moco) [2,20,21]. Moco-dependent enzymes perform various two-electron reduction-oxidation reactions, largely involving small polyatomic ions of carbon, nitrogen, and sulfur. The other ubiquitous pterin cofactor—H_4_folate (Figure 1B)—acts as a single-carbon carrier for multiple metabolic pathways and has been widely studied in the context of human health [22,23]. A biochemically related but structurally distinct one-carbon carrier cofactor—tetrahydromethanopterin (Figure 1B)—is present in methanogenic archaea [24,25], and methylotrophic bacteria also employ a related molecule for their one-carbon energy metabolism [26].

The ubiquity of pterin biosynthesis pathways coupled with the versatile chemical capabilities of these molecules enables them to fulfill diverse biological roles, many of which remain to be defined or fully understood. Recently, we discovered and characterized a novel function for pterins as signaling molecules in bacteria, regulating biofilm formation and likely other aspects of bacterial physiology [3,27,28]. In this review, we discuss the details of this pathway and connect to a broader theme of redox-dependent regulation as well as highlight key open questions in this emerging area.

## Pterins in bacterial biofilm formation

Biofilms are complex microbial communities that can provide constituent cells protection from the environment [29]. Many pathogens reside in biofilms as part of disease progression, persistence in spatially structured environments, and tolerance towards antibiotics [30]. Biofilm formation occurs as a progression of key steps [29]: (i) Cells approach a surface, either actively through motility or through passive processes. (ii) These surface-proximal cells make reversible interactions with the surface mediated by cell surface structures such as flagella and pili that bridge the boundary between cells and the surface. (iii) Stable surface attachment is mediated by the deployment of adhesin(s) that form a more durable and intimate connection. In many bacteria these adhesins are deployed in response to the surface, in addition to environmental regulation [31]. (iv) This attached population of cells proliferates and produces the biofilm matrix, mediating more cell-surface interaction and cell-to-cell cohesion, maturing into multiple layers and complex three-dimensional organization of cells. (v) At some point, due to a wide range of environmental signals, bacteria will disperse from the biofilm, re-entering a planktonic state [32]. Transitions between planktonic and sessile lifestyles are often regulated by chemical signals, including nutrient availability and stressors.

Many bacteria rely on signals to regulate where and when to establish biofilms, and among the most widely recognized signals is the cytoplasmic second messenger cyclic diguanylate monophosphate (c-di-GMP), which plays crucial regulatory roles in surface attachment, biofilm formation, motility, virulence, and the cell cycle [33]. Diguanylate cyclases (DGCs) drive synthesis of c-di-GMP from GTP, utilizing a canonical GGDEF catalytic motif. The signal molecule is broken down by phosphodiesterases (PDEs) that have either EAL or HD-GYP catalytic motifs [33]. Commonly, bacteria encode numerous GGDEF- and EAL-motif-containing proteins, and some have both catalytic motifs and can act as dual-function DGC-PDEs.

**Figure 2:**
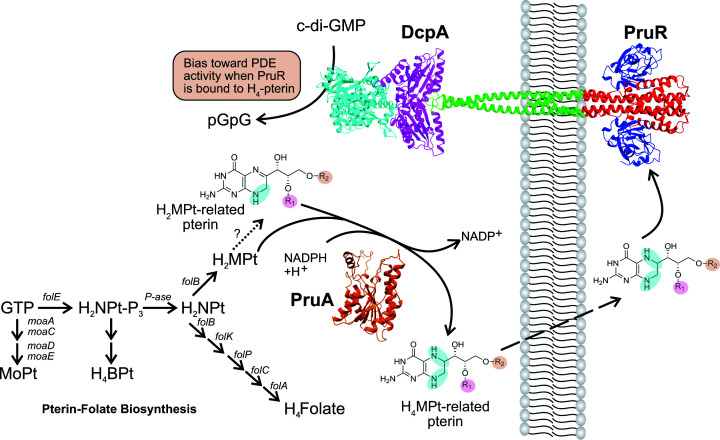
Model of pterin-dependent regulation of DcpA in *A. tumefaciens* Pterins are biosynthesized from GTP with GTP cyclohydrolase I (*folE*) catalyzing the first step in the folate, biopterin (BPt), and monapterin (MPt) biosynthesis pathways. Neopterin (NPt) and MPt are diastereomers (Figure 1B), differing in chirality at the 2′-hydroxyl position, and can be interconverted during or after the dephosphorylation of H_2_NPt-P_3_. The PruA pteridine reductase (orange) in the cytoplasm generates fully reduced H_4_MPt and/or H_4_MPt-related pterins that then enter the periplasm—via an unknown mechanism—where they bind to PruR (blue). The H_4_MPt-bound PruR interacts with the periplasmic portion of DcpA (red), inducing a conformational change that biases the enzymatic activity of DcpA toward a PDE (PDE domain shown in cyan and DGC domain shown in purple). This results in lower c-di-GMP concentrations in the cell, thus limiting biofilm formation. PruA and DcpA-PruR structure predictions were generated with AlphaFold, but the PruR structure alone has been crystallographically determined (PDB: 7KOS). P-ase = unidentified phosphatase. R_1_ = H or CH_3_; R_2_ = H or unknown sidechain.

The initial observation that pterins can influence biofilm formation came from studies on *Agrobacterium tumefaciens*, a pervasive proteobacterial (gram-negative or diderm) plant pathogen known for its ability to cause crown gall disease [34,35]. Crown gall is mediated by interkingdom DNA transfer, a process that has been extensively leveraged to genetically modify plants [35]. *Agrobacterium tumefaciens* utilizes the unipolar polysaccharide (UPP) adhesin to associate with biotic and abiotic surfaces, and form biofilms. *Agrobacterium tumefaciens* encodes numerous (>30) DGC and PDE homologs, several of which are dual-function DGC-PDEs, to regulate different processes, including biofilm formation [36]. One of the most impactful DGC-PDEs that controls surface attachment is regulated by pterins. The following sections will discuss the different components of this pterin-dependent regulatory pathway (Figure 2) and highlight the widespread nature of the genes encoding the key proteins involved, suggesting that pterins may function as signaling molecules in diverse bacteria.

## The pteridine reductase PruA negatively regulates surface attachment

Most biological pterins are synthesized from guanosine triphosphate (GTP) catalyzed by GTP cyclohydrolase I (*folE*), forming 7,8-dihydroneopterin triphosphate (H_2_NPt-P_3_). Various biosynthetic pathways then branch off from H_2_NPt-P_3_ to produce different pterin derivatives (Figure 2) [3,37]. The final functional pterin cofactors are the fully reduced H_4_pterin forms, including H_4_folate and tetrahydromonapterin (H_4_MPt); thus, dihydrofolate reductase (*folA*) and pteridine reductase enzymes act in the final biosynthesis steps to produce H_4_pterins from the respective H_2_pterins (Figure 1A). MoPt is synthesized from GTP in a separate pathway using MoaABCDE proteins [38,39] (Figure 2).

In 2013, we identified several genes that are important for the regulation of biofilm production in *A. tumefaciens*, including the surprising discovery of a pteridine reductase [40]. Transposon mutagenesis experiments coupled with analysis of UPP synthesis revealed that mutants in a gene for a putative pteridine reductase exhibit increased UPP production and biofilm formation [40]. The pteridine reductase belongs to the short-chain dehydrogenase/reductase (SDR) protein family and was named PruA (pteridine reductase A). Consistent with other pteridine reductases, PruA contains the conserved YX_3_K motif and the N-terminal TGX_3_RXG motif [41]. The underlined arginine of the N-terminal motif is conserved specifically among pteridine reductases, while the wider SDR family contains a glycine in its place [42]. PruA is homologous to FolM, a previously defined pteridine reductase in *E. coli* and other bacteria that catalyzes the reduction of dihydromonapterin (H_2_MPt) to H_4_MPt, which serves as a cofactor for amino acid hydroxylases such as phenylalanine hydroxylase [43].

Further experiments in *A. tumefaciens* demonstrated that a *pruA* deletion (Δ*pruA*), as well as a mutant producing a catalytically inactive enzyme, exhibit an overproduction of the adhesive polysaccharides, high levels of biofilm formation, and cellular aggregation [27]. Interestingly, pterin-profiling experiments in *A. tumefaciens* revealed the presence of a novel pterin species proposed to be 2′-*O*-methylmonapterin (2′-*O*-methyl-MPt), which is absent from the Δ*pruA* strain [27]. *In vitro* enzymatic experiments demonstrated that PruA serves as a H_2_MPt reductase to generate H_4_MPt [27,44], thus supporting a model in which H_4_MPt and/or 2′-*O*-methyl-H_4_MPt are required to negatively regulate biofilm formation in *A. tumefaciens* (Figure 2).

**Figure 3:**
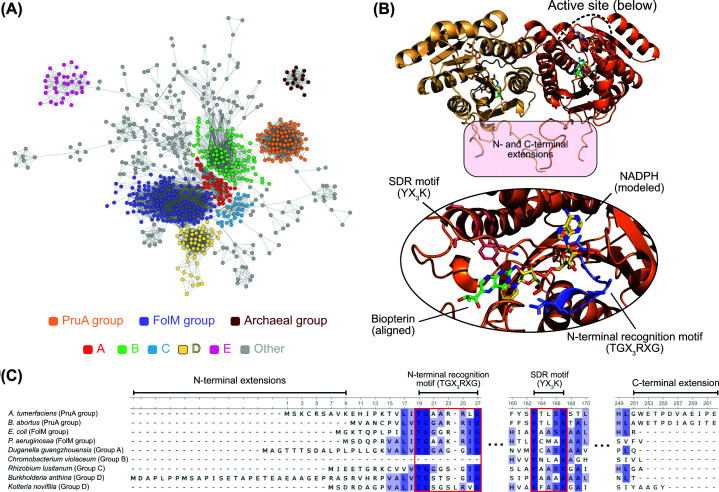
Sequence similarity and structural features across PruA homologs (**A**) SSN of bacterial and archaeal pteridine reductases. The EFI-Enzyme Similarity Tool [45,46] was used to generate a similarity network from protein sequences obtained from a BLAST search of *E. coli* FolM. Sequences were fetched with a negative log e-value of 2, consolidated into representative nodes of 40% identity, and connecting edges were trimmed down to 55% identity until groups were visually distinguishable in Cytoscape [63]. PruA and FolM groups were named according to their content of characterized pteridine reductases, while other sizeable and distinct groups were also distinguished by color. Small, disconnected groups were removed for visual clarity. The full network file is available in the Supplementary File. (**B**) An AlphaFold3 model of the *A. tumefaciens* PruA dimer with NADPH, aligned with biopterin bound to *L. major* PTR1 (PDB: 2BF7 [64]). N- and C-terminal extensions of the *A. tumefaciens* PruA modeled as low-confidence, unstructured regions. The active site is highlighted along with the YX_3_K and TGX_3_RXG motifs. (**C**) A multiple sequence alignment (MSA) of some representative sequences from the SSN. The alignment was generated in Unipro UGENE [65] with the MUSCLE algorithm [66]. Residues are numbered according to the *A. tumefaciens* PruA sequence. Interestingly, while the YX_3_K motif is broadly conserved as expected, a sequence from Group B lacks the N-terminal region altogether, while sequences from Groups A, C, and D have the TGX_3_GXG motif associated with the broader SDR family. Additionally, some sequences, including *A. tumefaciens* PruA, include N-terminal extensions. A C-terminal extension is also present in the *A. tumefaciens* and *B. abortus* PruAs.

Detailed enzymatic characterization of PruA demonstrated that the enzyme reduces H_2_pterins to their H_4_-forms, but does not reduce fully oxidized pterins [44]. Maximal catalytic efficiency was observed with dihydrobiopterin (H_2_BPt) as a substrate, and similar activities were obtained with dihydroneopterin (H_2_NPt) and H_2_MPt, while H_2_folate was not an effective substrate [44]. *Agrobacterium tumefaciens* lacks genes for biopterin biosynthesis or utilization, so H_2_BPt is unlikely to be the primary substrate for PruA. Given the lack of any known physiological function for neopterins beyond the role of H_2_NPt as an intermediate for folate biosynthesis (Figure 2), H_2_MPt is predicted to be the physiologically relevant substrate for PruA. The highly conserved PruA homolog from *Brucella abortus* (55% identity) exhibits a similar substrate profile to the *A. tumefaciens* PruA, while the *Pseudomonas aeruginosa* homolog (30% identity) has a markedly distinct substrate preference with maximal activity observed with H_2_folate [44]. A sequence similarity network (SSN) [45,46] of PruA/FolM-like proteins reveals that *E. coli* and *P. aeruginosa* pteridine reductases group together, while the *A. tumefaciens* and *B. abortus* enzymes constitute a distinct group (Figure 3A), which is overall consistent with experimentally observed substrate specificities. Along with the “FolM” and “PruA” type groups, several clusters comprised of uncharacterized SDRs arise in the SSN (Figure 3A). It remains unclear how broadly pteridine reductases function in signaling pathways in other bacteria, but the clustering of distinct groups may help inform future inquiry into pterin specificity and the associated physiological consequences.

## DcpA is a dual-function DCG-PDE regulated by pterins

DcpA—containing cytoplasmic DGC and a PDE domains, transmembrane segments, and a periplasmic domain (Figure 2)—was identified in the same genetic screen of *A. tumefaciens* that isolated mutants in the *pruA* pteridine reductase [40]. Deletion of *dcpA* (Δ*dcpA*) resulted in an increase in biofilm formation and UPP production, bypassing the normal surface contact dependence of UPP deployment [27]. Complementation of the *dcpA* mutant with wild-type *dcpA* or a GGDEF catalytic mutant (PDE-only) restored its normal biofilm and UPP phenotypes. In contrast, a PDE catalytic mutant of *dcpA* was unable to complement the ∆*dcpA* mutant and, in fact, strongly exacerbated its phenotypes [27]. These data suggested that DcpA is predominantly functioning as a PDE under laboratory conditions. Subsequent studies with the purified DcpA cytoplasmic domain confirmed both DGC and PDE enzymatic activities, and further characterization revealed that specific catalytic mutants retained either DGC or PDE activity, respectively [28]. Taken together, these studies established that DcpA is a dual-function DGC-PDE that limits c-di-GMP levels under laboratory conditions, preventing aberrant production of adhesin polysaccharides, and thereby restricting biofilm formation. Most relevantly, the PDE-biased state of DcpA requires PruA, thus linking c-di-GMP levels to the redox state of pterin metabolites [27] (Figure 2).

## PruR binds the PruA-dependent pterin signal and modulates DcpA activity

Immediately upstream and transcriptionally coupled to *dcpA* in *A. tumefaciens* is the *pruR* gene, encoding a pterin binding protein often annotated as a molybdopterin-dependent oxidoreductase, sharing weak sequence similarity with sulfite oxidase (SUOX) family enzymes such as *E. coli* YedY [47]. PruR is well-conserved across many *Proteobacteria* with similar domain and structural features, including N-terminal secretion signals, conserved SUOX motifs, and similar sizes (∼170 aa). Importantly, the PruR proteins lack the critical cysteine required to ligate the molybdenum of Moco found in enzymes such as in the SUOX family and instead have a tryptophan residue [28,48].

Deletion of *pruR* in *A. tumefaciens* resulted in an up-regulation of UPP and cellulose production, leading to biofilm formation, comparable to phenotypes of mutants lacking PruA activity [27,28]. Consistent with the presence of an N-terminal secretion signal, PruR was shown to be localized in the periplasm [28]. Notably, PruR interacts with the periplasmic but not the cytoplasmic portion of DcpA, and the interaction is dramatically diminished in the Δ*pruA* mutant [28]. *In vitro* analysis of PruR pterin binding revealed a significant preference for H_4_MPt over H_2_MPt, with generally less affinity for neopterin derivatives and folate [28], suggesting that the fully reduced pterin produced by PruA binds to PruR and promotes the PruR-DcpA interaction (Figure 2).

**Figure 4:**
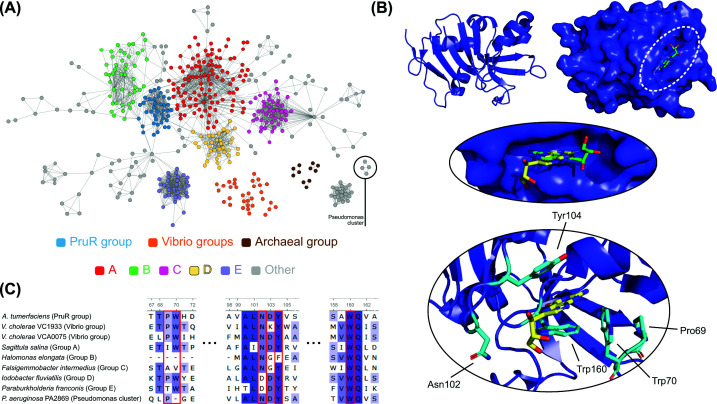
Sequence similarity and structural features across PruR homologs (**A**) SSN of bacterial and archaeal SUOX-like pterin binding proteins. The EFI-Enzyme Similarity Tool was used to generate a similarity network from protein sequences obtained from a BLAST search of *A. tumefaciens* PruR. The BLAST and SSN were created similarly to Figure 3, instead with a connecting edge identity cutoff of 57%. (**B**) The structure of *A. tumefaciens* PruR aligned with neopterin bound to *V. cholerae* PruR (PDBs: 7KOS, 7KP2, respectively [28]). The two opposite binding orientations of neopterin are shown. Notable and conserved active site residues are colored light blue. (**C**) An MSA of some representative sequences from the PruR SSN. The alignment was generated in Unipro UGENE with the MUSCLE algorithm. Residues are numbered according to the *A. tumefaciens* PruR sequence, and relevant residues are outlined in red. The tryptophan associated with pterin binding is mostly conserved, while the tyrosine is entirely conserved or conservatively replaced by phenylalanine in the Group B *Halomonas elongata*. The asparagine at position 102 (B,C) is substituted with an aspartate in Groups C and D sequences, potentially highlighting a specific mutation involved in pterin specificity.

The pterin-binding capability of PruR was confirmed through crystallographic structure determination, which revealed a SUOX-type fold with a truncated pterin binding site that accommodates smaller pterin species (Figure 4), but likely cannot accommodate the larger Moco moiety [28]. Taken together, this work indicates that PruR interacts with DcpA within the periplasm to modulate the cytoplasmic activities of DcpA and this interaction is stimulated in the presence of PruA-produced reduced pterins (Figure 2).

## Conservation of PruR-linked regulators in diverse proteobacteria

**Figure 5:**
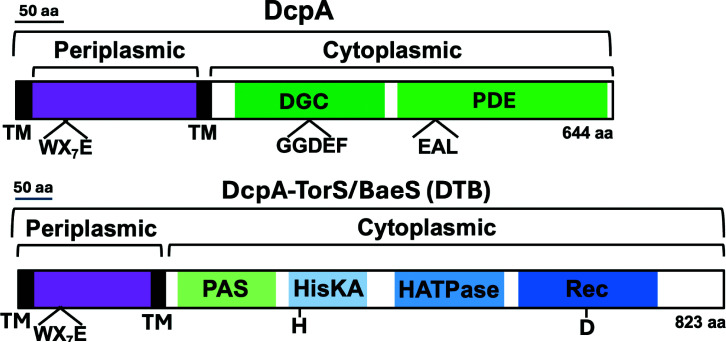
DcpA and DTB-type protein domain comparison DcpA protein from *A. tumefaciens* C58 (WP_006315771.1) and DTB-type protein from *Ruegeria pomeroyi* DSS-3 (WP_011045932.1). The periplasmic region (∼190 aa) and the WX_7_E motif it contains are conserved across these two classes. GGDEF and PDE catalytic motifs are indicated in the DcpA cytoplasmic region. The *R. pomeroyi* DTB-type protein cytoplasmic region has a PAS-type small ligand-binding domain, a two-component-type histidine kinase domain (HisKA), the histidine kinase ATPase domain (HATPase) to catalyze phosphorylation at the conserved histidine (H), and a C-terminal two-component-type receiver domain (Rec) with a conserved aspartate (D) that in other histidine kinases with Rec domains can be phosphorylated. Other DTB-type proteins can include all or a portion of these domains.

Bioinformatic probing of bacterial genomes for PruR homologs linked to genes encoding DcpA-type periplasmic domains revealed the conservation of PruR-DcpA gene pairs for a wide range of Proteobacteria [28]. Consolidating identical sequences and excluding a small number of pseudogenes compressed this set to >5500 unique sequences. The *pruR* and *dcpA* homologs identified are organized as a two-gene operon, as with the *A. tumefaciens* system. Strikingly, the vast majority have a strong match to a predicted periplasmic PruR and a DcpA-type periplasmic domain with a conserved WX_7_E motif. Among these regulatory pairs, there are two common configurations (Figure 5); one type is similar to that defined in *A. tumefaciens* with the DcpA-type periplasmic domain connected via a transmembrane segment to the c-di-GMP-related cytoplasmic domains. In the alternative configuration, the downstream regulator has a periplasmic domain similar to DcpA, but the cytoplasmic portion is a two-component sensor kinase domain [28]. This domain is generally most similar to TorS and BaeS sensor kinases, but can vary in a modular way between taxa, some with PAS and/or receiver domains, and these were named PruR-DTB (DcpA-TorS-BaeS) systems. The PruR-DcpA and PruR-DTB regulators are widely, but unevenly distributed within proteobacterial families, and are absent from some well-studied bacterial families (e.g., *Caulobacteriaceae*). Even so, the PruR-DcpA/DTB systems are broadly represented within Proteobacteria, and in a considerable number of single taxons they occur in multiple copies, with as many as four discrete systems, each with an operon encoding a PruR-type protein and either a DcpA or a DTB-type gene.

## Implications and future directions

### Extracellular pterins

From their earliest discovery in bacteria, it has become clear that pterins are excreted into the extracellular environment [49]. Comparisons of intracellular and extracellular pterin concentrations suggest significantly greater pterin concentrations outside than inside of bacterial cells [43]. Conversely, oxidized pterins can be supplied exogenously to compensate for folate pathway intermediates in specific mutants [50]. These observations on pterin excretion and uptake were always paradoxical, as the primary recognized activity of pterins in bacteria was to function as cofactors for cytoplasmic enzymes. Our findings that regulatory interactions for pterins occur in the periplasm via pterin-binding proteins and DcpA-type regulators now provide a discrete function for externalized pterins, although this is unlikely to be the primary reason that pterins are excreted. It is also currently unknown how pterins cross the membrane and whether this is a passive or active process. No pterin efflux systems have been identified, although there are bacteria with transporters for reduced folate derivatives [51]. The release of pterins into the extracellular environment allows for the possible exchange of these metabolites as signals between bacteria, as well as bacteria and host organisms. Furthermore, the redox-active nature of pterins may be important as an inherent regulatory mechanism, where reduced pterins are oxidized over time outside the cell, thus changing the interactions of these molecules with their signaling partners.

### Relevant pterins in PruR-DcpA signaling pathways

In *A. tumefaciens*, the PruA pteridine reductase is required to generate the most active pterin species for PruR-DcpA signaling [28]. However, the precise identity of the pterin molecule(s) that binds PruR *in vivo* remains unclear, including both the sidechain composition and the oxidation state of the pteridine ring. Our current findings suggest that the pterin species responsible for negatively regulating biofilm formation is an H_4_MPt derivative (Figure 2). It is possible that multiple pterins bind PruR to promote interaction with DcpA, and that distinct pterins induce different binding conformations that differentially modulate DcpA activity. Notably, the co-crystal structures of PruR with neopterin revealed three alternative binding conformations, with the molecule flipped in two opposite orientations [28] (Figure 4B). This could reflect that the oxidized neopterin used for crystallization is not the cognate binding partner and, thus, further studies are needed to identify the key pterin species involved in PruR-DcpA regulatory pathway.

Multiple bacteria lack a predicted pteridine reductase even though they have homologs to PruR and DcpA encoded in the characteristic two-gene operon. For example, *Vibrio cholerae* has two discrete PruR-DcpA regulatory pairs but does not have a PruA homolog to catalyze the reduction of H_2_pterins to the respective H_4_pterins [44]. Thus, it is unclear whether *V. cholerae* generates fully reduced pterins by another mechanism or instead does not utilize endogenously produced H_4_pterins. Another possible route to H_4_pterin species is via dihydrofolate reductase (DHFR)—this enzyme is generally thought to be highly specific for dihydrofolate; however, some DHFRs exhibit an expanded substrate scope and retain high activity with various six-substituted pterins [52]. In the absence of a *bona fide* pteridine reductase, it is plausible that *V. cholerae* may be tuned to exogenously produced pterins, and less so to the endogenous compounds. Interestingly, PruR-like proteins from *Vibrio* species cluster into several discrete groups that are separated from other organisms, suggesting that these regulatory systems are distinct (Figure 4A). Interrogation of PruR homologs from different organisms reveals the tryptophan associated with pterin binding (Trp160 in *A. tumefaciens* PruR) to be mostly conserved, while the tyrosine (Tyr104) facilitating pterin binding is most common but in some cases conservatively replaced by phenylalanine in some sequences (Figure 4B,C) [28]. Further studies are necessary to reveal the pterin-binding specificities of these different PruR-related proteins to determine their preference for different pterin oxidation states as well as the specific identities of the sidechains. Furthermore, the distribution of the PruA-FolM type pteridine reductases among different bacterial families—with and without PruR homologs—can help determine whether different systems are specific for reduced pterins produced by PruA-like enzymes.

### Diverse pterin structures with redox-dependent functions in bacteria

Novel pterin molecules continue to be identified in bacteria. A recent example includes the proposed antioxidant pterins known as colipterins, which have been discovered in probiotic, pathogenic, and commensal strains of *E. coli* [53] (Figure 1B). Colipterins were identified extracellularly, and production was up-regulated during treatment with subinhibitory concentrations of the antibiotic sulfamethoxazole (SMX), which targets the FolP enzyme in H_4_folate biosynthesis. Interestingly, in a colitis mouse model, colipterin ingestion prompted an anti-inflammatory immune response and significantly reduced colitis severity. The colipterins are derived from aromatic amino acids and monapterin, the latter of which is biosynthesized by a branch of the H_4_folate pathway prior to the SMX-inhibited FolP (Figure 2). Thus, the inhibition of H_4_folate synthesis results in metabolic rerouting to produce pterin metabolites that affect redox balance and host interactions [53].

In addition to serving as standalone small molecule antioxidants, the redox properties of pterins may provide a signaling cue to modulate the functions of downstream interacting partners to elicit a specific cellular response. A recent report described the interaction of several pteridine molecules with CutA, a cytoplasmic copper tolerance protein widely distributed from bacteria to eukaryotes [54]. When analyzed in untargeted experiments in cell extracts, CutA binds to 2′-deoxyxanthopterin B2, and *in vitro* experiments demonstrated interaction of CutA with dihydrobiopterin and H_4_BPt [54]. Notably, pterin-binding enhanced the ability of CutA to bind copper. Together, this work highlights the role of pterin metabolites as potential redox sensors to modulate metal homeostasis.

Other recent examples of pterins playing important new roles in bacteria are specifically associated with Moco. Bioinformatic prediction of genes involved in bacterial structural color (SC) revealed pterin pathway genes [55], and deletion experiments in *Flavobacterium* IR1 demonstrated that the lack of molybdopterin molybdenum transferase (*moeA*) resulted in SC shift and altered motility phenotypes when grown on different polysaccharide media [56]. Additionally, recent work in *Pseudomonas syringae* demonstrated that Moco is critical for socially induced motility [57]. In these examples, the pterin-dependent modulation of bacterial phenotypes may be a result of the traditional function of Moco as an enzymatic cofactor as opposed to pterins acting as signaling molecules. Thus, further work is necessary to reveal the mechanisms of these interesting Moco-dependent behaviors in bacteria.

Outside of pterins, the use of redox-active molecules as regulators in bacterial signaling pathways has established precedent. For example, in *Burkholderia thailandensis*, the heme-responsive protein NosP regulates the histidine kinase activity of NahK, where heme binding leads to biofilm suppression [58,59]. Quinones provide additional examples of redox-dependent regulation. In *E. coli*, the two-component regulatory system ArcA–AcrB is controlled by the redox states of ubiquinone pools to regulate the switch between anaerobic and aerobic energy metabolism [60]. Similar systems have been characterized in numerous bacteria that lead to regulatory outputs ranging from modulation of primary metabolism to biofilm formation and virulence [60,61]. Additionally, quinones modulate the activity of a diheme-dependent class III adenylate cyclase in *Sinorhizobium meliloti* [62]. In the quinone-regulated systems, the interacting protein has a redox active component (i.e., a disulfide bond or heme) that undergoes a redox reaction with the quinone/quinol, resulting in a conformational change that leads to the regulatory output. However, in the case of the pterin-dependent regulatory pathway, we do not have any evidence that the pterins chemically react with PruR. Instead, it appears to be a binding interaction that modulates interactions with DcpA (Figure 2).

## Concluding remarks

Pterins are versatile molecules with diverse roles in nature. The discovery of their involvement in a signaling pathway to control the motile-to-sessile switch in *A. tumefaciens* opens a new realm of pterin biochemistry for a regulatory mechanism widespread among diverse members of the Proteobacteria. The redox-active nature of pterins is expected to be a key aspect of different signaling pathways, where different oxidation states can serve as cues for various stressors and potentially facilitate distance-dependent intercellular cross-talk.

## Perspectives

- Bacterial biofilm formation is critically important in medical, industrial, and agricultural contexts, making its regulation a topic of significant microbiological interest. The discovery that pterins play a key role in regulating bacterial biofilms identifies a new potential target for intervention strategies and further expands the known scope of pterin-dependent biochemistry.
- The role of pterins in bacteria has traditionally been considered largely limited to their function as enzymatic cofactors. However, recent studies have expanded this view, highlighting pterins as potent regulatory molecules in bacteria. These findings have begun to elucidate the mechanisms underlying pterin-mediated regulation and suggest that these metabolites may serve as modulators of bacterial behavior.
- Much remains to be learned about how pterins influence regulatory processes in bacteria, including which specific pterins are relevant, including their redox state, how they transit the bacterial envelope, and how their interactions with pterin-binding proteins modulate the activity of associated regulators. Furthermore, the range of cellular functions influenced by pterins, as well as their potential roles as microbe-microbe and microbe-host signaling molecules, are areas of active investigation.

## Open Access

Open access for this article was enabled by the participation of Indiana University, Bloomington in an all-inclusive Read & Publish agreement with Portland Press and the Biochemical Society under an agreement with the Big Ten Alliance.

## Abbreviations

BPt: biopterin
c-di-GMP: cyclic-di-guanylate monophosphate
DHFR: dihydrofolate reductase
DGCs: Diguanylate cyclases
GTP: guanosine triphosphate
H^2^MPt: dihydromonapterin
H^2^NPt: dihydroneopterin
H^2^NPt-P^3^: dihydroneopterin triphosphate
H^4^folate: tetrahydrofolate
H^4^MPt: tetrahydromonapterin
Moco: molybdenum cofactor
MoPt: Molybdopterin
MPt: monapterin
MSA: multiple sequence alignment
NPt: Neopterin
PDEs: phosphodiesterases
PruA: pteridine reductase A
P-ase: phosphatase
SSN: sequence similarity network
SDR: short chain dehydrogenase/reductase
SC: structural color
SMX: sulfamethoxazole
SUOX: sulfite oxidase
UPP: unipolar polysaccharide
